# Fractional analysis of non-linear fuzzy partial differential equations by using a direct procedure

**DOI:** 10.1038/s41598-024-60123-5

**Published:** 2024-04-26

**Authors:** Muhammad Arshad, Shahbaz Khan, Hassan Khan, Hamid Ali, Ijaz Ali

**Affiliations:** 1 Department of Mathematics, Abdul Wali Khan University, Mardan, Pakistan; 2 Department of Mathematics, Near East University TRNC, Mersin 10, Turkey; 3 Department of Biosciences, COMSATS University Islamabad, Park Road Tarlai Kalan, Islamabad, 44000 Pakistan; 4 Centre for Applied Mathematics and Bioinformatics (CAMB), Gulf University for Science and Technology, 32093 Hawally, Kuwait

**Keywords:** Laplace transform, Power series, Fractional power series, Residual function, Fuzzy fractional partial differential equations, Applied mathematics, Computational science

## Abstract

In this study, an accurate analytical solution is presented for fuzzy FPDEs. It is done by using a novel method called the Laplace-residual power series (LRPSM) to build a series solution to the given problems. The fundamental instruments of the employed method are the Laplace transform, fractional Laurent, and fractional power series. Using the idea of a limit at infinity, we provide a series solution to a fuzzy FPDE with quick convergence and simple coefficient finding. We analyze three cases to obtain approximate and exact solutions to show the effectiveness and reliability of the Laplace- residual power series approach. To demonstrate the accuracy of the suggested procedure, we compare the findings to the real data.

## Introduction

Fractional calculus (FC) is very useful when modelling systems or processes that rely on inherited traits and preexisting ideas. A deeper and more thorough account of natural reality requires an understanding of fractional differential and integral calculus. It assists in predicting the future of the corresponding manifestations and modelling their early evolution. Furthermore, fractional differential and integral equations can convey more fascinating implications. Therefore, fractional calculus concentrates on phenomena that conventional calculus cannot model. Fractional differential equations (FDEs) are used to simulate a variety of physical phenomena, including magnetic and dynamical systems, engineering problems, biological and environmental concerns, and phenomena in the humanities^1–5^. Fractional derivatives are non-local in contrast to local integer derivatives, which are local. While integer-order derivatives can be used to explore changes in a point’s surroundings, fractional-order derivatives can be used to examine changes over the entire period. Systems with fractional order has gained a great deal of attention recently as an extension of the conventional order system.

### Related work

The Natural transforms of Prabhakar integral, Hilfer–Prabhakar (HP) fractional derivative and regularized Caputo form of HP fractional derivative (HPFD) are computed in^6^ by Dubay et al.. The author studied the Kharrat–Toma transforms of the Prabhakar integral, a Hilfer–Prabhakar (HP) fractional derivative, and the regularized version of the HP fractional derivative are derived and also compute the solution of some Cauchy problems and diffusion equations modeled with the HP fractional derivative via Kharrat–Toma transform^7^. Dubey et al. investigated the application of local fractional methods in combination with the local fractional Sumudu transform (LFST) for a local fractional Tricomi equation (LFTE)^8^. In Ref.^9^, the author extend the generalized invexity and duality results for multiobjective variational problems with fractional derivative pertaining to an exponential kernel by using the concept of weak minima. Partial differential equations (PDEs) may not always be the ideal choice when addressing real-world events. We must gather data from numerous sources to model dynamic systems. These data sets frequently lack certainty.In the modelling of such a system that lacks certainty, fuzzy set theory (FST) is used. Fuzzy fractional partial differential equations (FPDEs) became one of the fundamental themes of mainstream mathematical analysis as a result of the modelling of complex systems with a lack of certain data. As a result, several natural events are frequently described using fuzzy FPDEs concepts. Both FST and FC employ some computational techniques to enhance their comprehension of dynamic behaviour and lessen the uncertainty of their calculations. Finding exact analytical answers in the context of fuzzy FPDEs is a challenging procedure. A strong relationship exists between FST and FC. Due to its applicability to numerous scientific fields. In 1978, Kandel and Byatt introduced fuzzy DEs^10^. In contrast, fuzziness and the Riemann–Liouville (RL) differentiability idea were initially studied by Agarwal et al.^11^ using the Hukuhara-differentiability (HD) concept.

### Innovative contribution

Determining the exact solution to FPDEs is typically difficult due to the complexity of the model^12–14^. As a result, using numerical and analytical techniques to obtain an accurate solution is becoming increasingly popular, such as Kamal Shah et al. studied the solution of one-dimensional fuzzy FPDEs using a novel analytical technique^15^, For the system of fuzzy FDEs, Zahra et al. adopted the spline collecting method^16^, using the residual power series technique, Alaroud et al. explored specific types of fuzzy FDEs^17^, Khodadadi et al. investigated fuzzy FDEs with uncertainty by employing the variational iteration method^18^, Rahman et al. applied the fuzzy Sumudu transform to study fuzzy FDEs^19^, the nonlinear fuzzy partial differential equations are solved by Georgieva et al. by using the fuzzy Sawi decomposition method^20^, Alqurashi et al. examine the fuzzy nonlinear FPDEs via Elzaki transform^21^, Azim et al. used an extended differential transform approach to study fuzzy FDEs^22^, Stefania and Macías-Díaz use picard-like method for Caputo fuzzy FDEs^23^.

Dubey et al. used Sumudu residual power series method (SRPSM) to solve fractional Bloch equations appearing in an NMR flow^24^. In Ref.^25^, the authors used three different methods to study the investigation of fractional model of phytoplankton–toxic phytoplankton–zooplankton system with onvergence analysis. Dubey et al. the dynamics of atmospheric concentration of CO2 is investigated and studied through the application of a semi-analytical homotopy scheme combined with Sumudu transform and homotopy polynomials^26^. In Ref.^27^, a hepatitis E model involving a fractional derivative describing the viral dynamics of hepatitis E is explored and investigated via semi-analytical hybrid scheme pertaining to homotopy polynomials and the Sumudu transform algorithm.

In this manuscript, the Laplace-residual power series method (LRPSM)^28–32^ is implemented for obtaining the series solutions of non-linear fuzzy FPDEs. LRPSM hybridized the Laplace transform and RPSM^33–37^. The authors employed the limit rather than the derivative, as in RPSM, to obtain the coefficients in the series of Laurent and power, which speed up the process of doing so. Firstly, LT is applied on the targeted problem to convert the problem into algebraic equation and then the series solution is obtained by using Laurent and power series. In the end inverse LT is employed to get the desired results. Some of the advantages are mentioned below.

- The Laplace transform are used to tackle the fractional derivative, while using the other method such as RPSM, the fractional derivative are to be calculated in every step to find the series solution.
- The non-linearity is handled in a sophisticated manner as compare to other techniques such as LADM, HPM, NIM, and q-HAM.
- These existing techniques required more calculations to calculate the nonlinearity in each problem by using various polynomials such as Adomian and He’s polynomial.
- The solution are rapidly convergent to exact solution in most of the cases at integer order.

This paper is organized as follows: preliminaries of the Laplace transform, fuzzy set theory, and fuzzy fractional derivatives in the Caputo definition are expressed in “Preliminaries and theorems” section. The LRPS methodology for fuzzy FPDEs is proposed in “Laplace residual power series method (LRPSM) for non-linear fuzzy fractional partial differential equation (FPDE)” section. In “Numerical problem” section, the LRPSM procedure is implemented to solve non-linear fuzzy FPDEs. Finally, concluded the paper in “Conclusion” section.

## Preliminaries and theorems

This section contains some important FC and FST definitions and conclusions. For additional information, see^38,39^.

### **Definition 2.1**

Given a fuzzy function $\widetilde{v}$ continuous on [0, b] ⊂ R, then the integral of fuzzy corresponding to $t$ in the fractional RL-operator sense is defined as

$${\mathbf{I}}^{\boldsymbol{\alpha }}\widetilde{v}\left(t\right)={\int }_{o}^{t}\frac{{\left(t-\Phi \right)}^{\alpha -1}\widetilde{v}\left(\Phi \right)}{\Gamma (\alpha )}d\Phi ,\Phi \in \left(0,\infty \right).$$

Furthermore, fractional fuzzy integral is defined on $\widetilde{v}\in {\mathbb{C}}^{F}[0,\widetilde{b}]\cap {\mathbb{L}}^{F}[0,\widetilde{b}]$ where ${\mathbb{C}}^{F}[0,\widetilde{b}]$

is fuzzy functions space of continuous nature and ${\mathbb{L}}^{F}[0,\widetilde{b}]$ is integrable fuzzy Lebesgue functions space respectively;

$${[\mathbf{I}}^{\boldsymbol{\alpha }}\widetilde{v}\left(t\right){]}_{\eta }=\left[{{\mathbf{I}}^{\boldsymbol{\alpha }}{\underline{v}}_{\eta }\left(t\right),\mathbf{I}}^{\boldsymbol{\alpha }}{\overline{v} }_{\eta }\left(t\right)\right], 0\le \eta \le 1,$$

where

$${\mathbf{I}}^{\boldsymbol{\alpha }}{\underline{v}}\left(t\right)={\int }_{o}^{t}\frac{{\left(t-\Phi \right)}^{\alpha -1}\underline{v}\left(\Phi \right)}{\Gamma (\alpha )}d\Phi ,\Phi \in \left(0,\infty \right),$$

$${\mathbf{I}}^{\boldsymbol{\alpha }}\overline{v }\left(t\right)={\int }_{o}^{t}\frac{{\left(t-\Phi \right)}^{\alpha -1}\overline{v }\left(\Phi \right)}{\Gamma (\alpha )}d\Phi ,\Phi \in \left(0,\infty \right).$$

### **Definition 2.2**

Let $\widetilde{v}\in {\mathbb{C}}^{F}[0,b]\cap {\mathbb{L}}^{F}[0,b]$ in same line or a function, such that $[\widetilde{v}\left(t\right){]}_{\eta }=\left[{{\underline{v}}_{\eta }\left(t\right),}^{\boldsymbol{ }}{\overline{v} }_{\eta }\left(t\right)\right], \eta \in [\mathrm{0,1}]$ and ${t}_{0}\in (a,b)$, the fractional fuzzy derivative in Caputo sense is given as

$${[\mathbf{D}}^{\boldsymbol{\alpha }}\widetilde{v}\left(t\right){]}_{\eta }=\left[{{\mathbf{D}}^{\boldsymbol{\alpha }}{\underline{v}}_{\eta }\left(t\right),\mathbf{D}}^{\boldsymbol{\alpha }}{\overline{v} }_{\eta }\left(t\right)\right], 0<\alpha \le 1,$$

where

$${\mathbf{D}}^{\boldsymbol{\alpha }}{{\underline{v}}_{\eta }}\left({t}_{0}\right)={\left[{\int }_{o}^{t}\frac{{\left(t-\Phi \right)}^{r-\alpha -1}\frac{{d}^{r}}{d{\Phi }^{{\text{r}}}}\underline{v}\left(\Phi \right)}{\Gamma \left(r-\alpha \right)}d\Phi \right]}_{t={t}_{0}},$$

$${\mathbf{D}}^{\boldsymbol{\alpha }}{{\overline{v} }_{\eta }}\left({t}_{0}\right)={\left[{\int }_{o}^{t}\frac{{\left(t-\Phi \right)}^{r-\alpha -1}\frac{{d}^{r}}{d{\Phi }^{{\text{r}}}}{{\overline{v} }_{\eta }}\left(\Phi \right)}{\Gamma \left(r-\alpha \right)}d\Phi \right]}_{t={t}_{0}}.$$

Given that the integral converges and r = ⌈α⌉. Since α ∈ (0, 1], so r = 1.

### Theorem 1

Let u and $v$ be positive fuzzy numbers, then $w=u\odot v$ defined by $w\left(\eta \right)=\left[\underline{w}\left(\eta \right),\overline{w}\left(\eta \right)\right],$ where the following holds;

$$\underline{w}\left(\eta \right)=\underline{u}\left(\eta \right)\underline{v}\left(1\right)+\underline{u}\left(1\right)\underline{v}\left(\eta \right)-\underline{u}\left(1\right)\underline{v}\left(1\right),$$

And

$$\overline{w}\left(\eta \right)=\overline{u}\left(\eta \right)\overline{v}\left(1\right)+\overline{u}\left(1\right)\overline{v}\left(\eta \right)-\overline{u}\left(1\right)\overline{v}\left(1\right).$$

for every $\eta \in [\mathrm{0,1}]$ is a positive fuzzy number.

Let the set ${\mathbb{D}}$ be the domain of fuzzy-valued function $w$. Define the functions $\underline{w}\left(.,.,\eta \right), \overline{w} \left(.,.,\eta \right):{\mathbb{D}}\to {\mathbb{R}}\forall \eta \in \left[\mathrm{0,1}\right].$ These functions are said to be the left and right $\eta$-level functions of the function.

### Theorem 2

Let $$v:{\mathbb{R}}_{+}\to {\mathbb{E}}^{1}$$ and $$\forall \eta \in [\mathrm{0,1}]$$.

i. The function $\overline{v}\left(t,\eta \right) and \underline{v}(t,\eta )$ are Riemann-integrable on [0,b] for every $b\ge 0.$
ii. There are constant $\underline{M}\left(\eta \right)>0 and \overline{M}(\eta )>0$ such that the following holds:

$${\int }_{0}^{b}\left|\underline{v}\left(t,\eta \right)\right|dt\le \underline{M}\left(\eta \right), {\int }_{0}^{b}\left|\overline{v}\left(t,\eta \right)\right|dt\le \overline{M}\left(\eta \right),$$ for every $$b\ge 0.$$

Then, the function $v\left(t\right)$ is proper fuzzy Riemann-intgerable on $[0,\infty )$ and the following holds;

$$\left(FR\right){\int }_{0}^{\infty }v\left(t\right)dt=\left({\int }_{0}^{\infty }\underline{v}\left(t,\eta \right)dt,{\int }_{0}^{\infty }\overline{v}\left(t,\eta \right)dt\right).$$

### **Definition 2.3**

For $v(\zeta )$, LT of the fuzzy function is expressed as

$$v\left(s\right)={\mathcal{L}}_{t}\left[v\left(\zeta \right)\right]={\int }_{0}^{\infty }{e}^{-st}v\left(t\right)dt, t>0,$$

where $v\left(t\right)$ is the fuzzy valued function.

### **Definition 2.4**

LT of the convolution of fuzzy functions is defined as:

$${\mathcal{L}}_{t}\left[{v}_{1}*{v}_{2}\right]={\mathcal{L}}_{t}\left[{v}_{1}\right]*{\mathcal{L}}_{t}\left[{v}_{2}\right],$$

where ${v}_{1}*{v}_{2}$, is the fuzzy convolution of ${v}_{1}$ and ${v}_{2}$, i.e.

$${(v}_{1}*{v}_{2})={\int }_{0}^{t}{v}_{1}\left(t\right)*{v}_{2}\left(t-\varrho \right)d\varrho .$$

### **Definition 2.5**

Suppose that : ℜ → [0, 1] be a fuzzy number (FN) which satisfy the following properties^40^:

- $$v\left(\eta \zeta +\left(1-\eta y\right)\right)\ge {\text{min}}\left\{v\left(\zeta \right),v\left(y\right)\right\}, for \zeta ,y\in {\mathbb{R}},\eta \in \left[\mathrm{0,1}\right]$$.
- $$v\left(\zeta \right)=0, for some \left[\widetilde{w},\widetilde{v}\right].$$
- $v$ is increasing on $\left[\widetilde{w},\widetilde{a}\right]$ and decreasing on $\left[\widetilde{b},\widetilde{v}\right]$ having $\widetilde{w}\le \widetilde{a}\le \widetilde{b}\le \widetilde{v},$ and $v\left(\zeta \right)=1,$ for $\zeta \in \left[\widetilde{a},\widetilde{b}\right],$ where $\widetilde{a}, \widetilde{b}\in {\mathbb{R}}.$
- $v$ on ${\mathbb{R}}$ is upper semi-continuous.

${\mathbb{E}}^{1}$ represent the set of FNs. If $\widetilde{w}\in {\mathbb{R}},$ its represented as FN and ${\mathbb{R}}\subset {\mathbb{E}}^{1}.$

### **Definition 2.6**

The parametric formulation of the FN is as follows: $\left[{{\underline{v}}\left(\eta \right),}^{\boldsymbol{ }}{\overline{v} }\left(\eta \right)\right],$ such that $\eta \in [\mathrm{0,1}]$, together with the results:

- ${\overline{v} }\left(\eta \right)$ is bounded, right continuous, monotonically decreasing function on [0, 1].
- ${{\underline{v}}\left(\eta \right)}$ is left continuous, monotonically increasing and bounded function on [0, 1].
- $${{\underline{v}}\left(\eta \right)\le }^{\boldsymbol{ }}{\overline{v} }\left(\eta \right).$$

For FN $v=({{\underline{v}}\left(\eta \right),}^{\boldsymbol{ }}{\overline{v} }\left(\eta \right))$ and $\lambda \in {\mathbb{R}},$ then scalar multiplication and addition are defined as

$${\left[\lambda \odot v\right]}^{\eta }=\left\{\begin{array}{c}\left[\lambda {\underline{v}}\left(\eta \right){,\overline{v} }\left(\eta \right)\right], \lambda \ge 0,\\ \left[\lambda {\overline{v} }\left(\eta \right),{\underline{v}}\left(\eta \right)\right], \lambda <0.\end{array}\right.$$

and

$${\left[v\oplus \upsilon \right]}^{\eta }={\left[v\right]}^{\eta }+{\left[\upsilon \right]}^{\eta }=\left[{{\underline{v}}\left(\eta \right)+}^{\boldsymbol{ }}{\overline{v} }\left(\eta \right), \overline{\upsilon }\left(\eta \right)+{\underline{\upsilon }}(\eta )\right].$$

For the distance between FNs, authors used Hausdorff space.

### **Definition 2.7**

Let $v :{\mathbb{D}}\to {\mathbb{E}}^{1}$ and $\left({\zeta }_{0},t\right)\in {\mathbb{D}}.$ The mapping $v$ is called strongly generalized Hukuhara differentiable^41^ ($gH-differentiable$) on $({\zeta }_{0},t)$, if $\exists$ an element $\frac{\partial v\left({\zeta }_{0},t\right)}{\partial \zeta }\in {\mathbb{E}}^{1}$ then the following properties holds:

i. If $\forall \epsilon >0$ is sufficiently small, then the gH-differences exist
  $$v\left({\zeta }_{0}+\epsilon ,t\right){\ominus }_{gH} v\left({\zeta }_{0},t\right), v\left({\zeta }_{0},t\right){\ominus }_{gH} v\left({\zeta }_{0}+\epsilon ,t\right),$$
  and also the limits hold as:
  1 $$\underset{\epsilon \to 0}{{\text{lim}}}\frac{v\left({\zeta }_{0}+\epsilon ,t\right){\ominus }_{gH}v\left({\zeta }_{0},t\right)}{\epsilon } ,\underset{\epsilon \to 0}{{\text{lim}}}\frac{v\left({\zeta }_{0},t\right){\ominus }_{gH}v\left({\zeta }_{0}+\epsilon ,t\right)}{\epsilon }=\frac{\partial v\left({\zeta }_{0},t\right)}{\partial \zeta }$$
ii. If $$\forall \epsilon >0\; is \; reasonable \; small, \; then \; the \;gH{\text{-}}difference\; exist$$
  $$v\left({\zeta }_{0}-\epsilon ,t\right){\ominus }_{gH}v\left({\zeta }_{0},t\right), v\left({\zeta }_{0},t\right)\ominus v\left({\zeta }_{0}-\epsilon ,t\right),$$
  And also the limits hold as
  2 $$\underset{\epsilon \to 0}{{\text{lim}}}\frac{v\left({\zeta }_{0}-\epsilon ,t\right){\ominus }_{gH}v\left({\zeta }_{0},t\right)}{-\epsilon } ,\underset{\epsilon \to 0}{{\text{lim}}}\frac{v\left({\zeta }_{0},t\right){\ominus }_{gH}v\left({\zeta }_{0}-\epsilon ,t\right)}{-\epsilon }=\frac{\partial v\left({\zeta }_{0},t\right)}{\partial \zeta }$$

### Lemma 2.8(^42^)

Assume a fuzzy-valued continuous mapping $v:{\mathbb{D}}\to {\mathbb{E}}^{1}$ and $v\left(\zeta ,t\right)=\left[\underline{v}\left(\zeta ,t;\eta \right),\overline{v}\left(\zeta ,t;\eta \right)\right], \forall \eta \in \left[\mathrm{0,1}\right], thus$

i. Let $v(\zeta ,t)$ is (i)-differentiable for $\zeta$, then:
  3 $$\frac{\partial v\left({\zeta }_{0},t\right)}{\partial \zeta }=\left(\frac{\partial \underline{v}\left({\zeta }_{0},t\right)}{\partial \zeta },\frac{\partial \overline{v}\left({\zeta }_{0},t\right)}{\partial \zeta }\right).$$
ii. Let $v(\zeta ,t)$ is (ii)-differentiable for $\zeta$, then:
  4 $$\frac{\partial v\left({\zeta }_{0},t\right)}{\partial \zeta }=\left(\frac{\partial \overline{v}\left({\zeta }_{0},t\right)}{\partial \zeta },\frac{\partial \underline{v}\left({\zeta }_{0},t\right)}{\partial \zeta }\right).$$

## Laplace residual power series method (LRPSM) for non-linear fuzzy fractional partial differential equation (FPDE)

Here, we will discuss the methodology of LRPSM for the solution of fuzzy non-linear FPDE. The generalized form of fuzzy non-linear FPDE is presented as:

5 $$\sum_{i=0}^{k}{m}_{i}\odot {D}_{t}^{\alpha }v\left(,\zeta ,t\right)\oplus \sum_{j=1}^{f}{n}_{j}\odot \frac{{\partial }^{j}v\left(\zeta ,t\right)}{\partial {\zeta }^{j}}\oplus {\sum_{\psi =0}^{2}\sum_{\omega }^{2}{b}_{\psi \omega }\odot \frac{{\partial }^{\psi }v\left(\zeta ,t\right)}{\partial {\zeta }^{\psi }}\odot \frac{{\partial }^{\omega }v\left(\zeta ,t\right)}{\partial {\zeta }^{\omega }}=h\left(\zeta ,t\right), }$$

with initial condition (IC’s)

6 $$\frac{{\partial }^{i}u\left(\zeta ,0\right)}{\partial {\zeta }^{i}}={\vartheta }_{i}\left(\zeta \right), i=\mathrm{0,1}, \cdots ,k-1,$$

where $u, h:\left[0,b\right]\times \left[0,d\right]\to {\mathbb{E}}^{1}, {\vartheta }_{i}:\left[0,b\right]\to {\mathbb{E}}^{1}$ are fuzzy continuous mapping ${m}_{i}$, $i=\mathrm{1,2}, \cdots ,k; {n}_{j}, j=\mathrm{1,2},\cdots ,f, \psi =\mathrm{0,1},2,$ are non-negative constants.

Applying Laplace transform (LT) on Eq. (5),

7 $$\begin{aligned} & \sum_{i=0}^{k}{m}_{i}\odot {{\mathcal{L}}_{t}[D}_{t}^{\alpha }v\left(,\zeta ,t\right)]\oplus \sum_{j=1}^{f}{n}_{j}\odot {\mathcal{L}}_{t}\left({\mathcal{L}}_{t}^{-1}\left(\frac{{\partial }^{j}v\left(\zeta ,s\right)}{\partial {\zeta }^{j}}\right)\right) \\ & \quad \quad  \oplus {\sum_{\psi =0}^{2}\sum_{\omega }^{2}{b}_{\psi \omega }\odot {\mathcal{L}}_{t}\left({\mathcal{L}}_{t}^{-1}\left(\frac{{\partial }^{\psi }v\left(\zeta ,s\right)}{\partial {\zeta }^{\psi }}\right)\right)\odot {\mathcal{L}}_{t}\left({\mathcal{L}}_{t}^{-1}\left(\frac{{\partial }^{\omega }v\left(\zeta ,s\right)}{\partial {\zeta }^{\omega }}\right)\right)={\mathcal{L}}_{t}(h\left(\zeta ,t\right)), } \end{aligned}$$

and assume that $\frac{{\partial }^{\psi }v}{\partial {\zeta }^{\psi }}, \psi =\mathrm{0,1},2$, be fuzzy-valued positive functions, we get the parametric form as

8 $$ \begin{aligned} \sum_{i=0}^{k}{m}_{i}\odot {{\mathcal{L}}_{t}[D}_{t}^{\alpha }\underline{v}\left(,\zeta ,t\right)] & ={\mathcal{L}}_{t}\left(h\left(\zeta ,t\right)\right)-\sum_{j=1}^{f}{n}_{j}\odot {\mathcal{L}}_{t}\left({\mathcal{L}}_{t}^{-1}\left(\frac{{\partial }^{j}\underline{v}\left(\zeta ,s\right)}{\partial {\zeta }^{j}}\right)\right) \\ & \quad -{\sum_{\psi =0}^{2}\sum_{\omega }^{2}{b}_{\psi \omega }\odot {\mathcal{L}}_{t}\left({\mathcal{L}}_{t}^{-1}\left(\frac{{\partial }^{\psi }\underline{v}\left(\zeta ,s\right)}{\partial {\zeta }^{\psi }}\right)\right)\odot {\mathcal{L}}_{t}\left({\mathcal{L}}_{t}^{-1}\left(\frac{{\partial }^{\omega }\underline{v}\left(\zeta ,s\right)}{\partial {\zeta }^{\omega }}\right)\right), } \\ \sum_{i=0}^{k}{m}_{i}\odot {{\mathcal{L}}_{t}[D}_{t}^{\alpha }\overline{v}\left(,\zeta ,t\right)] & ={\mathcal{L}}_{t}\left(h\left(\zeta ,t\right)\right)-\sum_{j=1}^{f}{n}_{j}\odot {\mathcal{L}}_{t}\left({\mathcal{L}}_{t}^{-1}\left(\frac{{\partial }^{j}\overline{v}\left(\zeta ,s\right)}{\partial {\zeta }^{j}}\right)\right)  \\ & \quad -{\sum_{\psi =0}^{2}\sum_{\omega }^{2}{b}_{\psi \omega }\odot {\mathcal{L}}_{t}\left({\mathcal{L}}_{t}^{-1}\left(\frac{{\partial }^{\psi }\overline{v}\left(\zeta ,s\right)}{\partial {\zeta }^{\psi }}\right)\right)\odot {\mathcal{L}}_{t}\left({\mathcal{L}}_{t}^{-1}\left(\frac{{\partial }^{\omega }\overline{v}\left(\zeta ,s\right)}{\partial {\zeta }^{\omega }}\right)\right), } \end{aligned}$$

### **Case 1**

Consider the function $v(\zeta ,t;\eta )$ is $[\left(i\right)-\alpha ]$ partial differentiable of the $k-th$ order with respect to $t$ and $f$-th order with respect to $\zeta$.

Assume the Eq. (8), and using IC’s from Eq. (6), we have

9 $$\begin{aligned}\sum_{i=0}^{k}{m}_{i}[\underline{v}\left(,\zeta ,s\right)] & =\sum_{i=0}^{k}{m}_{i}\frac{1}{s}{\vartheta }_{0}\left(\zeta \right)+\frac{1}{{s}^{\alpha }}\left[{\mathcal{L}}_{t}\left(h\left(\zeta ,t\right)\right)\right]-\sum_{j=1}^{f}{n}_{j}\frac{1}{{s}^{\alpha }}{\mathcal{L}}_{t}\left({\mathcal{L}}_{t}^{-1}\left(\frac{{\partial }^{j}\underline{v}\left(\zeta ,s\right)}{\partial {\zeta }^{j}}\right)\right) \\ & \quad -{\sum_{\psi =0}^{2}\sum_{\omega }^{2}{b}_{\psi \omega }\frac{1}{{s}^{\alpha }}{\mathcal{L}}_{t}\left({\mathcal{L}}_{t}^{-1}\left(\frac{{\partial }^{\psi }\underline{v}\left(\zeta ,s\right)}{\partial {\zeta }^{\psi }}\right){\mathcal{L}}_{t}^{-1}\left(\frac{{\partial }^{\omega }\underline{v}\left(\zeta ,s\right)}{\partial {\zeta }^{\omega }}\right)\right), } \\ \sum_{i=0}^{k}{m}_{i}[\overline{v}\left(,\zeta ,s\right)] & =\sum_{i=0}^{k}{m}_{i}\frac{1}{s}{\vartheta }_{0}\left(\zeta \right)+\frac{1}{{s}^{\alpha }}\left[{\mathcal{L}}_{t}\left(h\left(\zeta ,t\right)\right)\right]-\sum_{j=1}^{f}{n}_{j}\frac{1}{{s}^{\alpha }}{\mathcal{L}}_{t}\left({\mathcal{L}}_{t}^{-1}\left(\frac{{\partial }^{j}\overline{v}\left(\zeta ,s\right)}{\partial {\zeta }^{j}}\right)\right) \\ & \quad -{\sum_{\psi =0}^{2}\sum_{\omega }^{2}{b}_{\psi \omega }\frac{1}{{s}^{\alpha }}{\mathcal{L}}_{t}\left({\mathcal{L}}_{t}^{-1}\left(\frac{{\partial }^{\psi }\overline{v}\left(\zeta ,s\right)}{\partial {\zeta }^{\psi }}\right){\mathcal{L}}_{t}^{-1}\left(\frac{{\partial }^{\omega }\overline{v}\left(\zeta ,s\right)}{\partial {\zeta }^{\omega }}\right)\right), } \end{aligned}$$

Assume that the solution of Eq. (9) has the following series

10 $$\begin{aligned}\underline{v}\left(\zeta ,s;\eta \right) & =\sum_{n=0}^{\infty }\frac{{{\underline{\vartheta }}}_{\eta }\left(\zeta \right)}{{s}^{n\alpha +1}}, \\ \overline{v}\left(\zeta ,s;\eta \right)&=\sum_{n=0}^{\infty }\frac{{\overline{\vartheta }}_{\eta }\left(\zeta \right)}{{s}^{n\alpha +1}}.\end{aligned}$$

And the j-th truncated term series are

11 $$\begin{aligned}\underline{v}\left(\zeta ,s;\eta \right)&=\frac{{\underline{\vartheta }}_{0}\left(\zeta \right)}{s}+\sum_{n=1}^{j}\frac{{\underline{\vartheta }}_{\eta }\left(\zeta \right)}{{s}^{n\alpha +1}}, \\ \overline{v}\left(\zeta ,s;\eta \right)&=\frac{{\overline{\vartheta }}_{0}}{s}+\sum_{n=1}^{j}\frac{{\overline{\vartheta }}_{\eta }\left(\zeta \right)}{{s}^{n\alpha +1}}.\end{aligned}$$

Laplace residual functions^28^ of Eq. (9) are given by

12 $$\begin{aligned}{\mathcal{L}}_{t}Re{s}_{\underline{v}}\left(\zeta ,s;\eta \right) & = \sum_{i=0}^{k}{m}_{i}[\underline{v}\left(,\zeta ,t\right)]-\sum_{i=0}^{k}{m}_{i}\frac{1}{s}{\vartheta }_{0}\left(\zeta \right)-\frac{1}{{s}^{\alpha }}\left[{\mathcal{L}}_{t}\left(h\left(\zeta ,t\right)\right)\right] \\ & \quad +\sum_{j=1}^{f}{n}_{j}\frac{1}{{s}^{\alpha }}{\mathcal{L}}_{t}\left({\mathcal{L}}_{t}^{-1}\left(\frac{{\partial }^{j}\underline{v}\left(\zeta ,s\right)}{\partial {\zeta }^{j}}\right)\right) \\ & \quad +{\sum_{\psi =0}^{2}\sum_{\omega }^{2}{b}_{\psi \omega }\frac{1}{{s}^{\alpha }}{\mathcal{L}}_{t}\left({\mathcal{L}}_{t}^{-1}\left(\frac{{\partial }^{\psi }\underline{v}\left(\zeta ,s\right)}{\partial {\zeta }^{\psi }}\right){\mathcal{L}}_{t}^{-1}\left(\frac{{\partial }^{\omega }\underline{v}\left(\zeta ,s\right)}{\partial {\zeta }^{\omega }}\right)\right), } \\ {\mathcal{L}}_{t}Re{s}_{\overline{v}}\left(\zeta ,s;\eta \right) &= \sum_{i=0}^{k}{m}_{i}[\overline{v}\left(\zeta ,t\right)]-\sum_{i=0}^{k}{m}_{i}\frac{1}{s}{\vartheta }_{0}\left(\zeta \right)-\frac{1}{{s}^{\alpha }}\left[{\mathcal{L}}_{t}\left(h\left(\zeta ,t\right)\right)\right] \\ & \quad +\sum_{j=1}^{f}{n}_{j}\frac{1}{{s}^{\alpha }}{\mathcal{L}}_{t}\left({\mathcal{L}}_{t}^{-1}\left(\frac{{\partial }^{j}\overline{v}\left(\zeta ,s\right)}{\partial {\zeta }^{j}}\right)\right) \\ & \quad +{\sum_{\psi =0}^{2}\sum_{\omega }^{2}{b}_{\psi \omega }\frac{1}{{s}^{\alpha }}{\mathcal{L}}_{t}\left({\mathcal{L}}_{t}^{-1}\left(\frac{{\partial }^{\psi }\overline{v}\left(\zeta ,s\right)}{\partial {\zeta }^{\psi }}\right){\mathcal{L}}_{t}^{-1}\left(\frac{{\partial }^{\omega }\overline{v}\left(\zeta ,s\right)}{\partial {\zeta }^{\omega }}\right)\right), }\end{aligned}$$

and the j-th Laplace residual functions are considered as

13 $$\begin{aligned}{\mathcal{L}}_{t}Re{s}_{\underline{v},j}\left(\zeta ,s;\eta \right) & = \sum_{i=0}^{k}{m}_{i}[{\underline{v}}_{j}\left(,\zeta ,t\right)]-\sum_{i=0}^{k}{m}_{i}\frac{1}{s}{\vartheta }_{0}\left(\zeta \right)-\frac{1}{{s}^{\alpha }}\left[{\mathcal{L}}_{t}\left(h\left(\zeta ,t\right)\right)\right] \\ & \quad +\sum_{j=1}^{f}{n}_{j}\frac{1}{{s}^{\alpha }}{\mathcal{L}}_{t}\left({\mathcal{L}}_{t}^{-1}\left(\frac{{\partial }^{j}{\underline{v}}_{j}\left(\zeta ,s\right)}{\partial {\zeta }^{j}}\right)\right) \\ & \quad +{\sum_{\psi =0}^{2}\sum_{\omega }^{2}{b}_{\psi \omega }\frac{1}{{s}^{\alpha }}{\mathcal{L}}_{t}\left({\mathcal{L}}_{t}^{-1}\left(\frac{{\partial }^{\psi }{\underline{v}}_{j}\left(\zeta ,s\right)}{\partial {\zeta }^{\psi }}\right){\mathcal{L}}_{t}^{-1}\left(\frac{{\partial }^{\omega }{\underline{v}}_{j}\left(\zeta ,s\right)}{\partial {\zeta }^{\omega }}\right)\right), } \\ {\mathcal{L}}_{t}Re{{s}_{\overline{v}}}_{j}\left(\zeta ,s;\eta \right) & = \sum_{i=0}^{k}{m}_{i}[{\overline{v}}_{j}\left(,\zeta ,t\right)]-\sum_{i=0}^{k}{m}_{i}\frac{1}{s}{\vartheta }_{0}\left(\zeta \right)-\frac{1}{{s}^{\alpha }}\left[{\mathcal{L}}_{t}\left(h\left(\zeta ,t\right)\right)\right] \\ & \quad +\sum_{j=1}^{f}{n}_{j}\frac{1}{{s}^{\alpha }}{\mathcal{L}}_{t}\left({\mathcal{L}}_{t}^{-1}\left(\frac{{\partial }^{j}{\overline{v}}_{j}\left(\zeta ,s\right)}{\partial {\zeta }^{j}}\right)\right) \\ & \quad +{\sum_{\psi =0}^{2}\sum_{\omega }^{2}{b}_{\psi \omega }\frac{1}{{s}^{\alpha }}{\mathcal{L}}_{t}\left({\mathcal{L}}_{t}^{-1}\left(\frac{{\partial }^{\psi }{\overline{v}}_{j}\left(\zeta ,s\right)}{\partial {\zeta }^{\psi }}\right){\mathcal{L}}_{t}^{-1}\left(\frac{{\partial }^{\omega }{\overline{v}}_{j}\left(\zeta ,s\right)}{\partial {\zeta }^{\omega }}\right)\right), }\end{aligned}$$

Here some properties of Laplace residual functions are presented, which are important in obtaining the analytical solution are given as

- $${\mathcal{L}}_{t}Re{s}\left(\zeta ,s;\eta \right)=0 and \underset{j\to \infty }{{\text{lim}}}{\mathcal{L}}_{t}Re{s}_{{\underline{v}}_{j}}\left(\zeta ,s;\eta \right)={\mathcal{L}}_{t}Re{s}_{{\underline{v}}_{j}}\left(\zeta ,s;\eta \right) for each s>0.$$
- $${\mathcal{L}}_{t}Re{s}\left(\zeta ,s;\eta \right)=0 and \underset{j\to \infty }{{\text{lim}}}{\mathcal{L}}_{t}Re{s}_{{\overline{v}}_{j}}\left(\zeta ,s;\eta \right)={\mathcal{L}}_{t}Re{s}_{{\overline{v}}_{j}}\left(\zeta ,s;\eta \right) for each s>0.$$
- $${\underset{s\to \infty }{{\text{lim}}}s\mathcal{L}}_{t}Re{s}_{\underline{v}}\left(\zeta ,s;\eta \right)=0\Rightarrow {\underset{s\to \infty }{{\text{lim}}}s\mathcal{L}}_{t}Re{s}_{{\underline{v}}_{j}}\left(\zeta ,s;\eta \right)=0.$$
- $${\underset{s\to \infty }{{\text{lim}}}s\mathcal{L}}_{t}Re{s}_{\overline{v}}\left(\zeta ,s;\eta \right)=0\Rightarrow {\underset{s\to \infty }{{\text{lim}}}s\mathcal{L}}_{t}Re{s}_{{\overline{v}}_{j}}\left(\zeta ,s;\eta \right)=0.$$
- $${\underset{s\to \infty }{{\text{lim}}}{s}^{j\alpha +1}\mathcal{L}}_{t}Re{s}_{\underline{v}}\left(\zeta ,s;\eta \right)={\underset{s\to \infty }{{\text{lim}}}{s}^{j\alpha +1}\mathcal{L}}_{t}Re{s}_{{\underline{v}}_{j}}\left(\zeta ,s;\eta \right)=0, j=\mathrm{1,2},3,\cdots .$$
- $${\underset{s\to \infty }{{\text{lim}}}{s}^{j\alpha +1}\mathcal{L}}_{t}Re{s}_{\overline{v}}\left(\zeta ,s;\eta \right)={\underset{s\to \infty }{{\text{lim}}}{s}^{j\alpha +1}\mathcal{L}}_{t}Re{s}_{{\overline{v}}_{j}}\left(\zeta ,s;\eta \right)=0, j=\mathrm{1,2},3,\cdots .$$

For obtaining the terms ${\underline{\vartheta }}_{n}(\zeta )$ and ${\overline{\vartheta }}_{n}(\zeta )$, we solve recursively the following system:

14 $$\begin{aligned} & {\underset{s\to \infty }{{\text{lim}}}{s}^{j\alpha +1}\mathcal{L}}_{t}Re{s}_{{\underline{v}}_{j}}\left(\zeta ,s;\eta \right)=0, j=\mathrm{1,2},3,\cdots , \\ & {\underset{s\to \infty }{{\text{lim}}}{s}^{j\alpha +1}\mathcal{L}}_{t}Re{s}_{{\overline{v}}_{j}}\left(\zeta ,s;\eta \right)=0, j=\mathrm{1,2},3,\cdots \end{aligned}$$

In the end, applying inverse LT to Eq. (11), we obtain the j-th approximate solutions of $\underline{v}\left(\zeta ,s;\eta \right)$ and $\overline{v}\left(\zeta ,s;\eta \right)$.

### Case 2

Consider the function $v\left(\zeta ,s;\eta \right)$ is $[\left(ii\right)-\alpha ]$ differentiable of the 2 $k-th$ order with respect to $t$ and [(i)-$\alpha$]-differentiable of $f$-th order with respect to $\zeta$, then the parametric equation of Eq. (7) is

15 $$\begin{aligned} \sum_{i=0}^{k}{m}_{2i}{{\mathcal{L}}_{t}[D}_{t}^{2\alpha }\underline{v}\left(,\zeta ,t\right)]+\sum_{i=0}^{k}{m}_{2i-1}{{\mathcal{L}}_{t}[D}_{t}^{\alpha }\underline{v}\left(,\zeta ,t\right)]& ={\mathcal{L}}_{t}\left(h\left(\zeta ,t\right)\right)-\sum_{j=1}^{f}{n}_{j}{\mathcal{L}}_{t}\left({\mathcal{L}}_{t}^{-1}\left(\frac{{\partial }^{j}\underline{v}\left(\zeta ,s\right)}{\partial {\zeta }^{j}}\right)\right) \\ & \quad -{\sum_{\psi =0}^{2}\sum_{\omega }^{2}{b}_{\psi \omega }{\mathcal{L}}_{t}\left({\mathcal{L}}_{t}^{-1}\left(\frac{{\partial }^{\psi }\underline{v}\left(\zeta ,s\right)}{\partial {\zeta }^{\psi }}\right){\mathcal{L}}_{t}^{-1}\left(\frac{{\partial }^{\omega }\underline{v}\left(\zeta ,s\right)}{\partial {\zeta }^{\omega }}\right)\right), } \\ \sum_{i=0}^{k}{m}_{2i}{{\mathcal{L}}_{t}[D}_{t}^{2\alpha }\overline{v}\left(,\zeta ,t\right)]+\sum_{i=0}^{k}{m}_{2i-1}{{\mathcal{L}}_{t}[D}_{t}^{\alpha }\overline{v}\left(,\zeta ,t\right)]&={\mathcal{L}}_{t}\left(h\left(\zeta ,t\right)\right)-\sum_{j=1}^{f}{n}_{j}{\mathcal{L}}_{t}\left({\mathcal{L}}_{t}^{-1}\left(\frac{{\partial }^{j}\overline{v}\left(\zeta ,s\right)}{\partial {\zeta }^{j}}\right)\right) \\ & \quad -{\sum_{\psi =0}^{2}\sum_{\omega }^{2}{b}_{\psi \omega }{\mathcal{L}}_{t}\left({\mathcal{L}}_{t}^{-1}\left(\frac{{\partial }^{\psi }\overline{v}\left(\zeta ,s\right)}{\partial {\zeta }^{\psi }}\right){\mathcal{L}}_{t}^{-1}\left(\frac{{\partial }^{\omega }\overline{v}\left(\zeta ,s\right)}{\partial {\zeta }^{\omega }}\right)\right), }\end{aligned}$$

Using definition of Laplace transform for fuzzy valued function and IC’s, we get

16 $$\begin{aligned}{\varvec{A}}{\mathcal{L}}_{t}\left[\underline{v}\left(,\zeta ,t\right)\right]+{\varvec{B}}{\mathcal{L}}_{t}\left[\underline{v}\left(,\zeta ,t\right)\right] & ={\mathbb{G}}_{1}\left(\zeta ,\eta \right)-{\mathcal{L}}_{t}\left(h\left(\zeta ,t\right)\right)-\sum_{j=1}^{f}{n}_{j}{\mathcal{L}}_{t}\left({\mathcal{L}}_{t}^{-1}\left(\frac{{\partial }^{j}\underline{v}\left(\zeta ,s\right)}{\partial {\zeta }^{j}}\right)\right) \\ & \quad -{\sum_{\psi =0}^{2}\sum_{\omega }^{2}{b}_{\psi \omega }{\mathcal{L}}_{t}\left({\mathcal{L}}_{t}^{-1}\left(\frac{{\partial }^{\psi }\underline{v}\left(\zeta ,s\right)}{\partial {\zeta }^{\psi }}\right){\mathcal{L}}_{t}^{-1}\left(\frac{{\partial }^{\omega }\underline{v}\left(\zeta ,s\right)}{\partial {\zeta }^{\omega }}\right)\right), } \\ {\varvec{A}}{{\mathcal{L}}_{t}[}\overline{v}\left(,\zeta ,t\right)]+{\varvec{B}}{{\mathcal{L}}_{t}[}\overline{v}\left(,\zeta ,t\right)] & ={\mathbb{G}}_{2}\left(\zeta ,\eta \right)-{\mathcal{L}}_{t}\left(h\left(\zeta ,t\right)\right)-\sum_{j=1}^{f}{n}_{j}{\mathcal{L}}_{t}\left({\mathcal{L}}_{t}^{-1}\left(\frac{{\partial }^{j}\overline{v}\left(\zeta ,s\right)}{\partial {\zeta }^{j}}\right)\right) \\ & \quad -  {\sum_{\psi =0}^{2}\sum_{\omega }^{2}{b}_{\psi \omega }{\mathcal{L}}_{t}\left({\mathcal{L}}_{t}^{-1}\left(\frac{{\partial }^{\psi }\overline{v}\left(\zeta ,s\right)}{\partial {\zeta }^{\psi }}\right){\mathcal{L}}_{t}^{-1}\left(\frac{{\partial }^{\omega }\overline{v}\left(\zeta ,s\right)}{\partial {\zeta }^{\omega }}\right)\right), }\end{aligned}$$

where $${\varvec{A}}=\sum_{i=0}^{k}{m}_{2i}{s}^{2\alpha },{\varvec{B}}=\sum_{i=0}^{k}{m}_{2i-1}{s}^{\alpha }, {\mathbb{G}}_{1}\left(\zeta ,\eta \right)=\sum_{i=0}^{k}{m}_{2i}\left({s}^{1-2\alpha }{\underline{\vartheta }}_{0}\left(\zeta ,\eta \right)+{s}^{2-2\alpha }{\overline{\vartheta }}_{1}\left(\zeta ,\eta \right)\right)+\sum_{i=0}^{k}{m}_{2i-1}\left({s}^{1-2\alpha }{\overline{\vartheta }}_{0}\left(\zeta ,\eta \right)+{s}^{2-2\alpha }{\underline{\vartheta }}_{1}\left(\zeta ,\eta \right)\right)$$ and $${\mathbb{G}}_{2}\left(\zeta ,\eta \right)=\sum_{i=0}^{k}{m}_{2i}\left({s}^{1-2\alpha }{\overline{\vartheta }}_{0}\left(\zeta ,\eta \right)+{s}^{2-2\alpha }{\underline{\vartheta }}_{1}\left(\zeta ,\eta \right)\right)+\sum_{i=0}^{k}{m}_{2i-1}\left({s}^{1-2\alpha }{\underline{\vartheta }}_{0}\left(\zeta ,\eta \right)+{s}^{2-2\alpha }{\overline{\vartheta }}_{1}\left(\zeta ,\eta \right)\right).$$

From the above system, we find $\underline{v}\left(\zeta ,s;\eta \right)$ and $\overline{v}\left(\zeta ,s;\eta \right)$, similar to Case 1, we obtain $v\left(\zeta ,t\right)=(\underline{v}\left(\zeta ,s;\eta \right) ,\overline{v}\left(\zeta ,s;\eta \right)).$

## Numerical problem

LRPSM is illustrated by discussing the following problems.

### Problem

Suppose the fuzzy FPDE as^20^

17 $${D}_{t}^{2\alpha }v\left(\zeta ,t\right)\oplus \frac{\partial v\left(\zeta ,t\right)}{\partial \zeta }\odot \frac{{\partial }^{2}v\left(\zeta ,t\right)}{\partial {\zeta }^{2}}=h\left(\zeta ,t\right), \zeta >0, t\ge 0, 0<\alpha \le 1,$$

Subject to the IC’s

18 $$v\left(\zeta ,0\right)=\left(\frac{{\zeta }^{2}}{2}\eta ,\frac{{\zeta }^{2}}{2}(2-\eta )\right), {v}_{t}\left(\zeta ,0\right)=\left(\mathrm{0,0}\right), \zeta >0,$$

where $$h\left(\zeta ,t\right)=\left(\eta +\zeta {\eta }^{2},2-\eta +\zeta {\left(2-\eta \right)}^{2}\right).$$

Now, for the solution of Eq. (17), we will discuss three cases.

#### Case 1

Let $$v\left(\zeta ,t\right) is \left[\left(i\right)-\alpha \right]-differentiable.$$

Applying the LT on Eq. (17), and putting IC’s from Eq. (18)

19 $$\begin{aligned}\underline{v}\left(\zeta ,s;\eta \right) & =\frac{1}{s}\frac{{\zeta }^{2}}{2}\eta -\frac{1}{{s}^{2\alpha }}{\mathcal{L}}_{t}\left[{\mathcal{L}}_{t}^{-1}\left(\frac{\partial \underline{v}\left(\zeta ,t\right)}{\partial \zeta }\right){\mathcal{L}}_{t}^{-1}\left(\frac{{\partial }^{2}\underline{v}\left(\zeta ,t\right)}{\partial {\zeta }^{2}}\right)\right]+\frac{1}{{s}^{2\alpha } }{\mathcal{L}}_{t}\left[h\left(\zeta ,t;\eta \right)\right], \\ \overline{v}\left(\zeta ,s;\eta \right) & =\frac{1}{s}\frac{{\zeta }^{2}}{2}\left(2-\eta \right)-\frac{1}{{s}^{2\alpha }}{\mathcal{L}}_{t}\left[{\mathcal{L}}_{t}^{-1}\left(\frac{\partial \overline{v}\left(\zeta ,t\right)}{\partial \zeta }\right){\mathcal{L}}_{t}^{-1}\left(\frac{{\partial }^{2}\overline{v}\left(\zeta ,t\right)}{\partial {\zeta }^{2}}\right)\right]+\frac{1}{{s}^{2\alpha } }{\mathcal{L}}_{t}\left[h\left(\zeta ,t;\eta \right)\right].\end{aligned}$$

The ${j}$th-truncated term series form solution of Eq. (19) are given by

20 $$\begin{aligned} \underline{v}\left(\zeta ,s;\eta \right)&=\frac{1}{s}\frac{{\zeta }^{2}}{2}\eta +\sum_{i=1}^{j}\frac{{\underline{\vartheta }}_{i}\left(\zeta \right)}{{s}^{(1+i)\alpha +1}}, \\ \overline{v}\left(\zeta ,s;\eta \right)&=\frac{1}{s}\frac{{\zeta }^{2}}{2}\left(2-\eta \right)+\sum_{i=1}^{j}\frac{{\overline{\vartheta }}_{i}\left(\zeta \right)}{{s}^{(1+i)\alpha +1}}. \end{aligned}$$

and the ${j}$th-Laplace residual lower and upper functions are given respectively

21 $$\begin{aligned} {\mathcal{L}}_{t}Re{s}_{{\underline{v}}_{j}}& =\underline{v}\left(\zeta ,s;\eta \right)-\frac{1}{s}\frac{{\zeta }^{2}}{2}\eta +\frac{1}{{s}^{2\alpha }}{\mathcal{L}}_{t}\left[{\mathcal{L}}_{t}^{-1}\left(\frac{\partial \underline{v}\left(\zeta ,t\right)}{\partial \zeta }\right){\mathcal{L}}_{t}^{-1}\left(\frac{{\partial }^{2}\underline{v}\left(\zeta ,t\right)}{\partial {\zeta }^{2}}\right)\right]-\frac{1}{{s}^{2\alpha } }{\mathcal{L}}_{t}(\eta +\zeta {\eta }^{2}), \\ {\mathcal{L}}_{t}Re{s}_{{\overline{v}}_{j}}& =\overline{v}\left(\zeta ,s;\eta \right)-\frac{1}{s}\frac{{\zeta }^{2}}{2}\left(2-\eta \right)+\frac{1}{{s}^{2\alpha }}{\mathcal{L}}_{t}\left[{\mathcal{L}}_{t}^{-1}\left(\frac{\partial \overline{v}\left(\zeta ,t\right)}{\partial \zeta }\right){\mathcal{L}}_{t}^{-1}\left(\frac{{\partial }^{2}\overline{v}\left(\zeta ,t\right)}{\partial {\zeta }^{2}}\right)\right]-\frac{1}{{s}^{2\alpha } }{\mathcal{L}}_{t}(2-\eta +\zeta (2-\eta )^2).\end{aligned}$$

To solve the above system, we substitute the Eq. (20) of ${j}$th-truncated term series into ${j}$th-Laplace residual functions of Eq. (21), and then the obtained result are multiplied by ${s}^{\left(i+1\right)\alpha +1}$ and the relations ${\underset{s\to \infty }{{\text{lim}}}{s}^{(i+1)\alpha +1}\mathcal{L}}_{t}Re{s}_{{\underline{v}}_{j}}\left(\zeta ,s;\eta \right)=0$ and ${\underset{s\to \infty }{{\text{lim}}}{s}^{(i+1)\alpha +1}\mathcal{L}}_{t}Re{s}_{{\overline{v}}_{j}}\left(\zeta ,s;\eta \right)=0$ are solved recursively, we obtain the following terms of lower and upper solutions respectively,

22 $$\begin{aligned}{\underline{\vartheta }}_{0}\left(\zeta ,\eta \right)& =\frac{{\zeta }^{2}}{2}\eta , \\ {\overline{\vartheta }}_{0}\left(\zeta , \eta \right)& =\frac{{\zeta }^{2}}{2}\left(2-\eta \right), \\ {\underline{\vartheta }}_{1}\left(\zeta ,\eta \right) & =\eta , \\ {\overline{\vartheta }}_{1}\left(\zeta , \eta \right) & =\left(2-\eta \right), \\ {\underline{\vartheta }}_{2}\left(\zeta ,\eta \right) & =0, \\ {\overline{\vartheta }}_{2}\left(\zeta , \eta \right) & =0, \\ {\underline{\vartheta }}_{3}\left(\zeta ,\eta \right) & =0, \\ {\overline{\vartheta }}_{3}\left(\zeta , \eta \right)&=0, \\ \vdots \end{aligned} $$

Putting the values of ${\underline{\vartheta }}_{i}\left(\zeta ,\eta \right)$ and ${\overline{\vartheta }}_{i}\left(\zeta , \eta \right)$, $i=\mathrm{1,2},3,\dots$ in Eq. (20), we get

23 $$\begin{aligned} \underline{v}\left(\zeta ,s;\eta \right) & =\frac{1}{s}\frac{{\zeta }^{2}}{2}\eta +\frac{\eta }{{s}^{2\alpha +1}}+0+0+\cdots , \\ \overline{v}\left(\zeta ,s;\eta \right)& =\frac{1}{s}\frac{{\zeta }^{2}}{2}\left(2-\eta \right)+\frac{(2-\eta )}{{s}^{2\alpha +1}}+0+0+\cdots . \end{aligned}$$

Employing inverse LT on Eq. (23), we have

24 $$\begin{aligned}\underline{v}\left(\zeta ,t;\eta \right) & =\frac{{\zeta }^{2}}{2}\eta +\frac{\eta }{\Gamma (2\alpha +1)}{t}^{2\alpha }, \\ \overline{v}\left(\zeta ,t;\eta \right) & =\frac{{\zeta }^{2}}{2}\left(2-\eta \right)+\frac{(2-\eta )}{\Gamma (2\alpha +1)}{t}^{2\alpha }. \end{aligned}$$

The solution is presented as follows

25 $$v\left(\zeta ,t\right)=\left(\left(\frac{{\zeta }^{2}}{2}+\frac{{t}^{2\alpha }}{\Gamma \left(2\alpha +1\right)}\right)\eta ,\left(\frac{{\zeta }^{2}}{2}+\frac{{t}^{2\alpha }}{\Gamma \left(2\alpha +1\right)}\right)(2-\eta )\right).$$

#### Case 2

Let $v(\zeta ,t)$ is $\left[\left(ii\right)-\alpha \right]-differentiable.$ From Eq. (16), we obtain

26 $$\begin{aligned} \underline{v}\left(\zeta ,s;\eta \right)&=\frac{{\underline{\vartheta }}_{0}(\zeta ;\eta )}{s}-\frac{1}{{s}^{2\alpha }}{\mathcal{L}}_{t}\left[{\mathcal{L}}_{t}^{-1}\left(\frac{\partial \underline{v}\left(\zeta ,t\right)}{\partial \zeta }\right){\mathcal{L}}_{t}^{-1}\left(\frac{{\partial }^{2}\underline{v}\left(\zeta ,t\right)}{\partial {\zeta }^{2}}\right)\right]+\frac{1}{{s}^{2\alpha } }{\mathcal{L}}_{t}\left[h\left(\zeta ,t;\eta \right)\right], \\ \overline{v}\left(\zeta ,s;\eta \right)& =\frac{{\overline{\vartheta }}_{0}(\zeta ;\eta )}{s}-\frac{1}{{s}^{2\alpha }}{\mathcal{L}}_{t}\left[{\mathcal{L}}_{t}^{-1}\left(\frac{\partial \overline{v}\left(\zeta ,t\right)}{\partial \zeta }\right){\mathcal{L}}_{t}^{-1}\left(\frac{{\partial }^{2}\overline{v}\left(\zeta ,t\right)}{\partial {\zeta }^{2}}\right)\right]+\frac{1}{{s}^{2\alpha } }{\mathcal{L}}_{t}\left[h\left(\zeta ,t;\eta \right)\right]. \end{aligned}$$

Thus, Case 2 is similar to Case 1.

#### Case 3

If $v\left(\zeta ,t\right)$ is $\left[\left(i\right)-\alpha \right]-{\text{differentiable}}$ and ${v}_{t}(\zeta ,t)$ is [(ii)-$\alpha$]-differentiable w.r.t t respectively, Thus

27 $${\mathcal{L}}_{t}\left({v}{\prime}\left(\zeta ,t\right)\right)=\left[{\mathcal{L}}_{t}\left({\underline{v}}{\prime}\left(\zeta ,t\right)\right),{\mathcal{L}}_{t}\left({\overline{v}}{\prime}\left(\zeta ,t\right)\right)\right],$$

and

28 $${\mathcal{L}}_{t}\left({v}^{{\prime}{\prime}}\left(\zeta ,t\right)\right)=\left[{\mathcal{L}}_{t}\left({\underline{v}}^{{\prime}{\prime}}\left(\zeta ,t\right)\right),{\mathcal{L}}_{t}\left({\overline{v}}^{{\prime}{\prime}}\left(\zeta ,t\right)\right)\right].$$

Applying the LT on Eq. (17), and putting IC’s from Eq. (18)

29 $$\begin{aligned}\underline{v}\left(\zeta ,s;\eta \right) & =\frac{1}{s}\frac{{\zeta }^{2}}{2}\eta -\frac{1}{{s}^{2\alpha }}{\mathcal{L}}_{t}\left[{\mathcal{L}}_{t}^{-1}\left(\frac{\partial \overline{v}\left(\zeta ,t\right)}{\partial \zeta }\right){\mathcal{L}}_{t}^{-1}\left(\frac{{\partial }^{2}\overline{v}\left(\zeta ,t\right)}{\partial {\zeta }^{2}}\right)\right]+\frac{1}{{s}^{2\alpha } }{\mathcal{L}}_{t}\left[\overline{h}\left(\zeta ,t;\eta \right)\right], \\ \overline{v}\left(\zeta ,s;\eta \right) & =\frac{1}{s}\frac{{\zeta }^{2}}{2} (2-\eta )-\frac{1}{{s}^{2\alpha }}{\mathcal{L}}_{t}\left[{\mathcal{L}}_{t}^{-1}\left(\frac{\partial \underline{v}\left(\zeta ,t\right)}{\partial \zeta }\right){\mathcal{L}}_{t}^{-1}\left(\frac{{\partial }^{2}\underline{v}\left(\zeta ,t\right)}{\partial {\zeta }^{2}}\right)\right]+\frac{1}{{s}^{2\alpha } }{\mathcal{L}}_{t}\left[\underline{h}\left(\zeta ,t;\eta \right)\right]. \end{aligned}$$

The ${j}$th-truncated term series form solution of Eq. (29) are given by

30 $$\begin{aligned} \underline{v}\left(\zeta ,s;\eta \right) & =\frac{1}{s}\frac{{\zeta }^{2}}{2}\eta +\sum_{i=1}^{j}\frac{{\underline{\vartheta }}_{i}\left(\zeta \right)}{{s}^{(1+i)\alpha +1}}, \\ \overline{v}\left(\zeta ,s;\eta \right) & =\frac{1}{s}\frac{{\zeta }^{2}}{2}\left(2-\eta \right)+\sum_{i=1}^{j}\frac{{\overline{\vartheta }}_{i}\left(\zeta \right)}{{s}^{(1+i)\alpha +1}}.\end{aligned}$$

and the ${j}$th-Laplace residual lower and upper functions are given respectively

31 $$\begin{aligned} {\mathcal{L}}_{t}Re{s}_{{\underline{v}}_{j}} & =\underline{v}\left(\zeta ,s;\eta \right)-\frac{1}{s}\frac{{\zeta }^{2}}{2}\eta +\frac{1}{{s}^{2\alpha }}{\mathcal{L}}_{t}\left[{\mathcal{L}}_{t}^{-1}\left(\frac{\partial \overline{v}\left(\zeta ,t\right)}{\partial \zeta }\right){\mathcal{L}}_{t}^{-1}\left(\frac{{\partial }^{2}\overline{v}\left(\zeta ,t\right)}{\partial {\zeta }^{2}}\right)\right]+\frac{1}{{s}^{2\alpha } }{\mathcal{L}}_{t}\left[\overline{h}\left(\zeta ,t;\eta \right)\right], \\ {\mathcal{L}}_{t}Re{s}_{{\overline{v}}_{j}} & =\overline{v}\left(\zeta ,s;\eta \right)-\frac{1}{s}\frac{{\zeta }^{2}}{2} \left(2-\eta \right)+\frac{1}{{s}^{2\alpha }}{\mathcal{L}}_{t}\left[{\mathcal{L}}_{t}^{-1}\left(\frac{\partial \underline{v}\left(\zeta ,t\right)}{\partial \zeta }\right){\mathcal{L}}_{t}^{-1}\left(\frac{{\partial }^{2}\underline{v}\left(\zeta ,t\right)}{\partial {\zeta }^{2}}\right)\right]+\frac{1}{{s}^{2\alpha } }{\mathcal{L}}_{t}\left[\underline{h}\left(\zeta ,t;\eta \right)\right].\end{aligned}$$

To solve the above system, we substitute the Eq. (30) of ${j}$th-truncated term series into ${j}$th-Laplace residual functions of Eq. (31), and then the obtained result are multiplied by ${s}^{\left(i+1\right)\alpha +1}$ and the relations ${\underset{s\to \infty }{{\text{lim}}}{s}^{(i+1)\alpha +1}\mathcal{L}}_{t}Re{s}_{{\underline{v}}_{j}}\left(\zeta ,s;\eta \right)=0$ and ${\underset{s\to \infty }{{\text{lim}}}{s}^{(i+1)\alpha +1}\mathcal{L}}_{t}Re{s}_{{\overline{v}}_{j}}\left(\zeta ,s;\eta \right)=0$ are solved recursively, we obtain the following terms of lower and upper solutions respectively,

32 $$\begin{aligned} {\underline{\vartheta }}_{0}\left(\zeta ,\eta \right) & =\frac{{\zeta }^{2}}{2}\eta , \\ {\overline{\vartheta }}_{0}\left(\zeta , \eta \right) & =\frac{{\zeta }^{2}}{2}\left(2-\eta \right), \\ {\underline{\vartheta }}_{1}\left(\zeta ,\eta \right) & =2-\eta , \\ {\overline{\vartheta }}_{1}\left(\zeta , \eta \right) & =\eta , \\ {\underline{\vartheta }}_{2}\left(\zeta ,\eta \right)& =0, \\ {\overline{\vartheta }}_{2}\left(\zeta , \eta \right) & =0, \\ {\underline{\vartheta }}_{3}\left(\zeta ,\eta \right)& =0, \\ {\overline{\vartheta }}_{3}\left(\zeta , \eta \right) & =0, \\ \vdots \end{aligned}  $$

Putting the values of ${\underline{\vartheta }}_{i}\left(\zeta ,\eta \right)$ and ${\overline{\vartheta }}_{i}\left(\zeta , \eta \right)$, $i=\mathrm{1,2},3,\dots$ in Eq. (30), we get

33 $$\begin{aligned}\underline{v}\left(\zeta ,s;\eta \right) & =\frac{1}{s}\frac{{\zeta }^{2}}{2}\eta +\frac{(2-\eta )}{{s}^{2\alpha +1}}+0+0+\cdots , \\ \overline{v}\left(\zeta ,s;\eta \right) & =\frac{1}{s}\frac{{\zeta }^{2}}{2}\left(2-\eta \right)+\frac{\eta }{{s}^{2\alpha +1}}+0+0+\cdots .\end{aligned}$$

Employing inverse LT on Eq. (34), we have

34 $$\begin{aligned}\underline{v}\left(\zeta ,t;\eta \right) & =\frac{{\zeta }^{2}}{2}\eta +\frac{(2-\eta )}{\Gamma (2\alpha +1)}{t}^{2\alpha }, \\ \overline{v}\left(\zeta ,t;\eta \right) & =\frac{{\zeta }^{2}}{2}\left(2-\eta \right)+\frac{\eta }{\Gamma (2\alpha +1)}{t}^{2\alpha }.\end{aligned}$$

The solution is presented as follows

35 $$v\left(\zeta ,t\right)=\left(\left(\frac{{\zeta }^{2}\eta }{2}+\frac{(2-\eta ){t}^{2\alpha }}{\Gamma \left(2\alpha +1\right)}\right),\left(\frac{{\zeta }^{2}(2-\eta )}{2}+\frac{\eta {t}^{2\alpha }}{\Gamma \left(2\alpha +1\right)}\right)\right).$$

## Results and discussions

**Figure 1:**
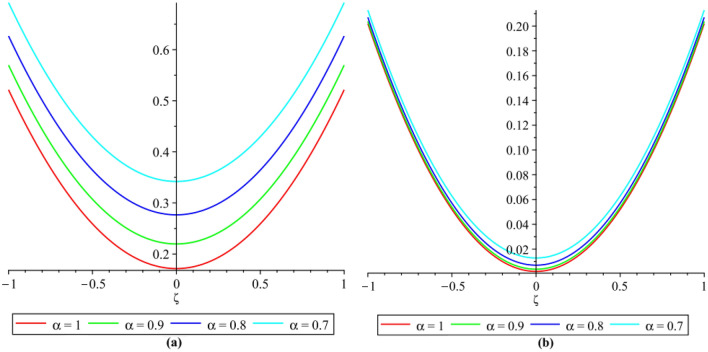
2D plots of the Case 1 lower solutions at (**a**) $\eta$ = 0.7 and t = 0.7, (**b**) $\eta$ = 0.4 and t = 0.1 and various values of α.

**Figure 2:**
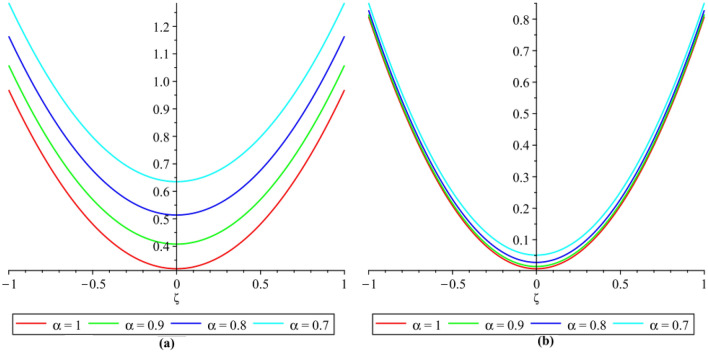
2D plots of the Case 1 upper solutions at (**a**) $\eta$ = 0.7 and t = 0.7, (**b**) $\eta$ = 0.4 and t = 0.1 and various values of α.

**Figure 3:**
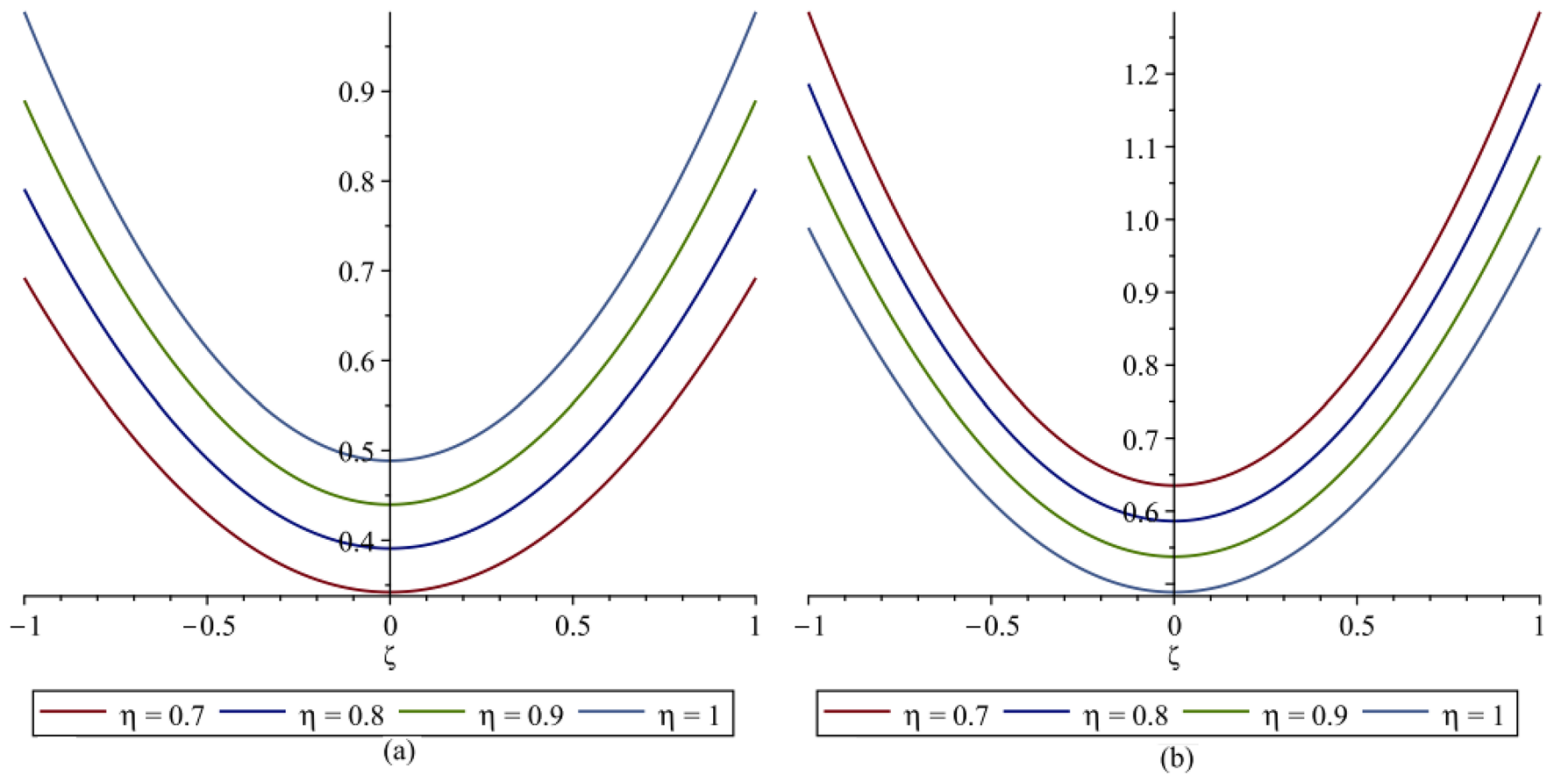
2D plots of the Case 1 lower and upper solutions at different values of $\eta$, t = 0.7 and α = 0.7.

**Figure 4:**
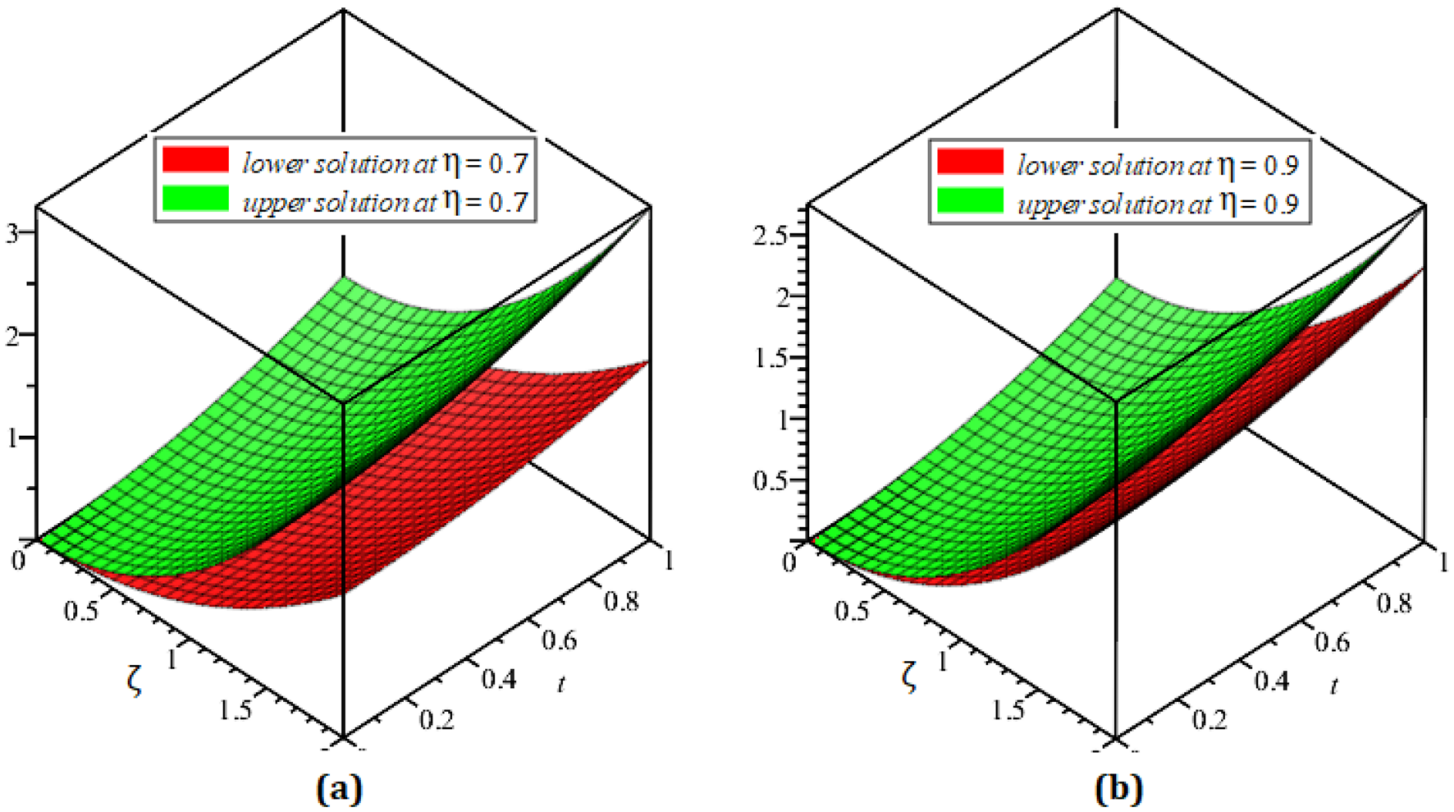
3D plots of the Case 1 lower and upper solutions at (**a**) $\eta$ = 0.7, (**b**) $\eta$ = 0.9 and α = 1.

**Figure 5:**
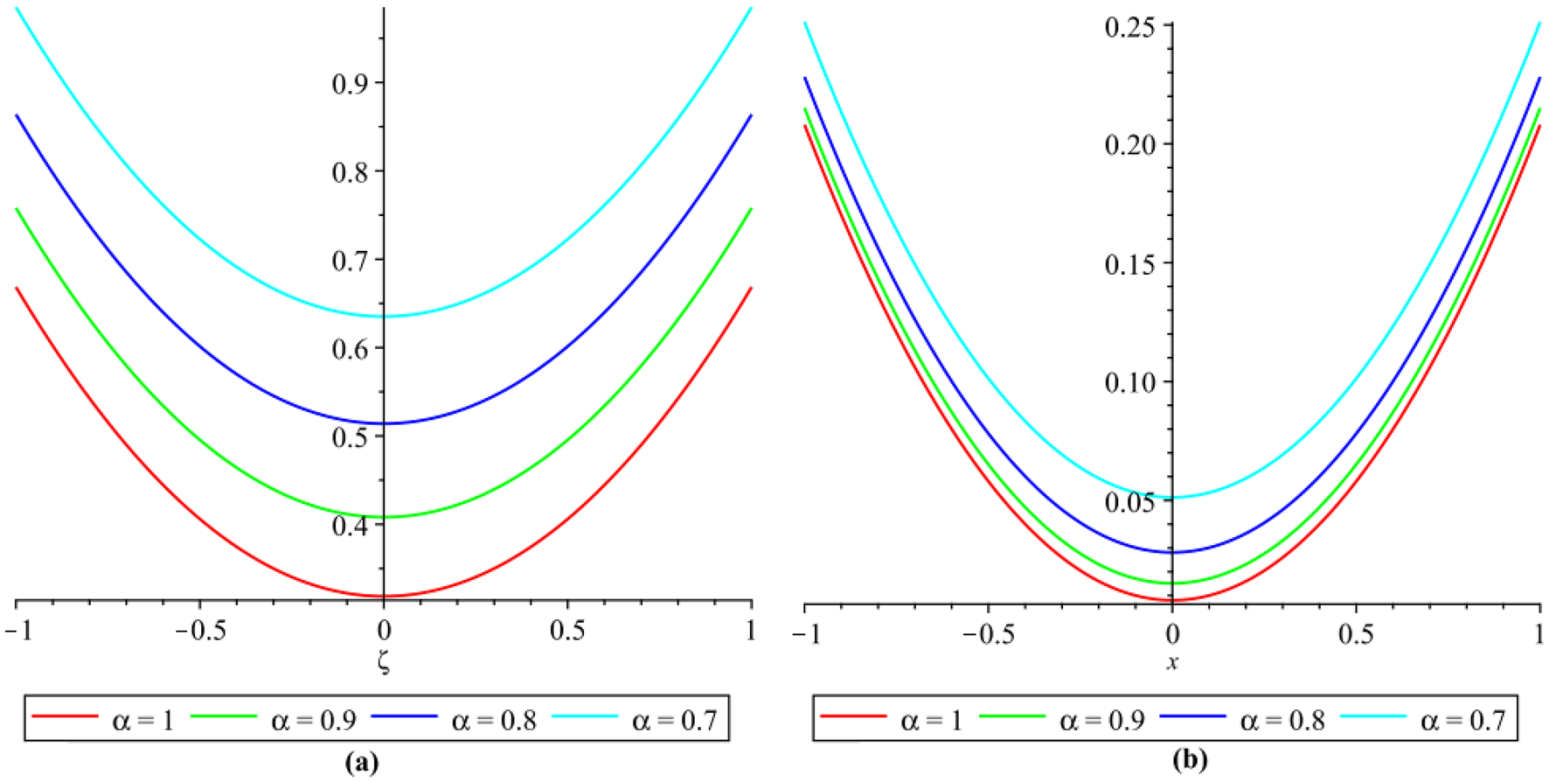
2D plots of the Case 3 lower solutions at (**a**) $\eta$ = 0.7, t = 0.7, (**b**) $\eta$ = 0.4, t = 0.7 and various values of α.

**Figure 6:**
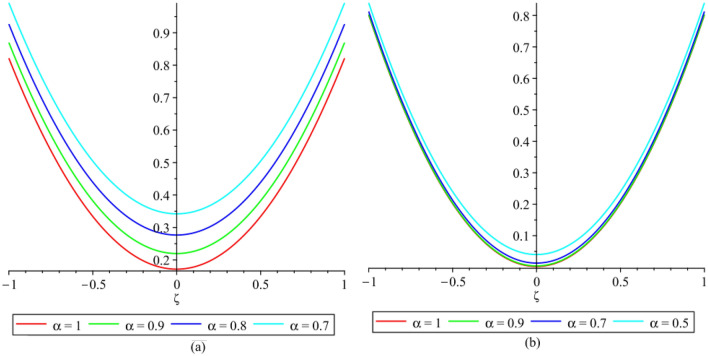
2D plots of the Case 3 upper solutions at (**a**) $\eta$ = 0.7, t = 0.7, (**b**) $\eta$ = 0.4, t = 0.1 and various values of α.

**Figure 7:**
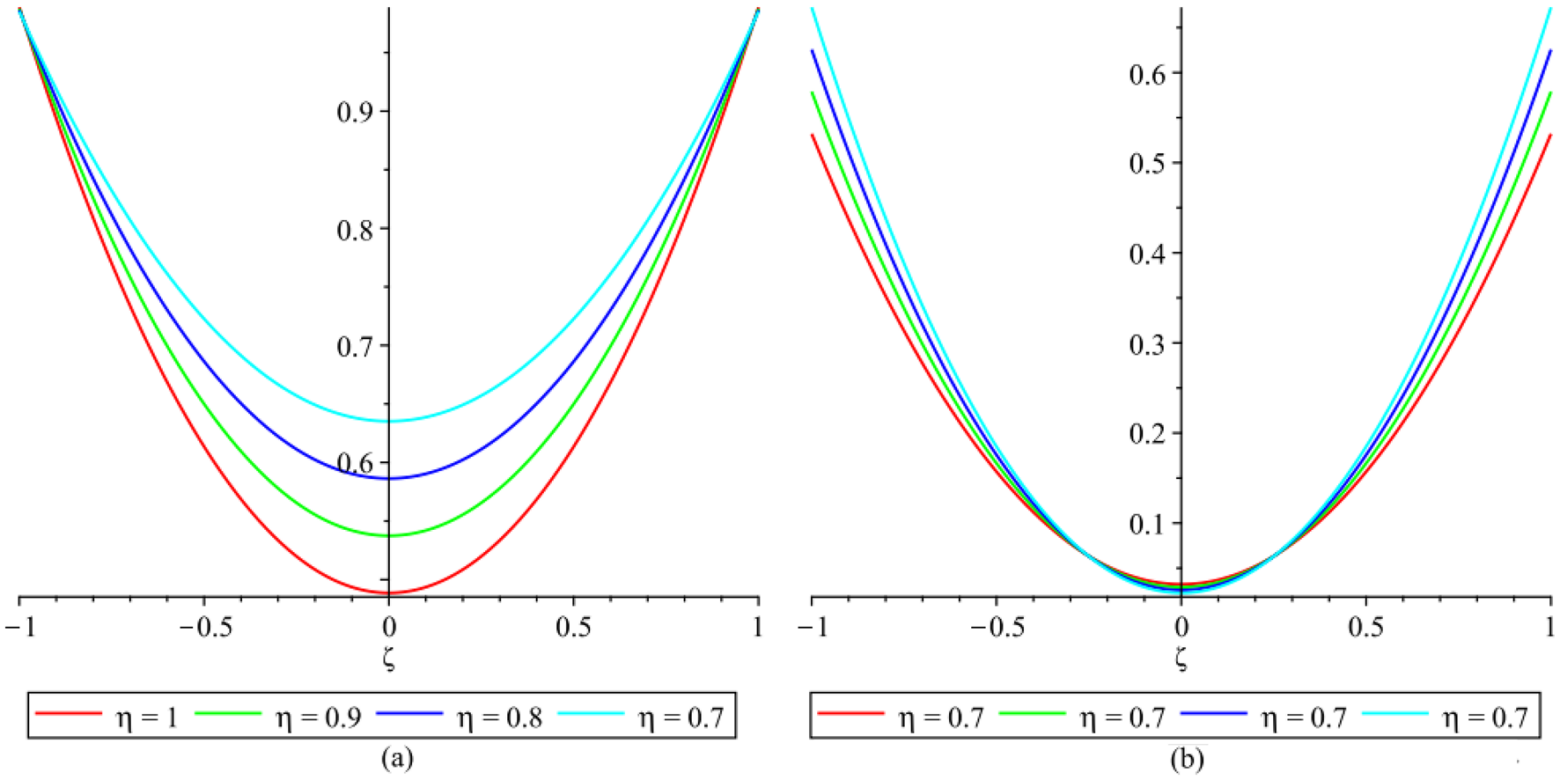
2D plots of the Case 3 lower and upper solutions at various values of $\eta$, t = 0.1 and α = 0.7.

**Figure 8:**
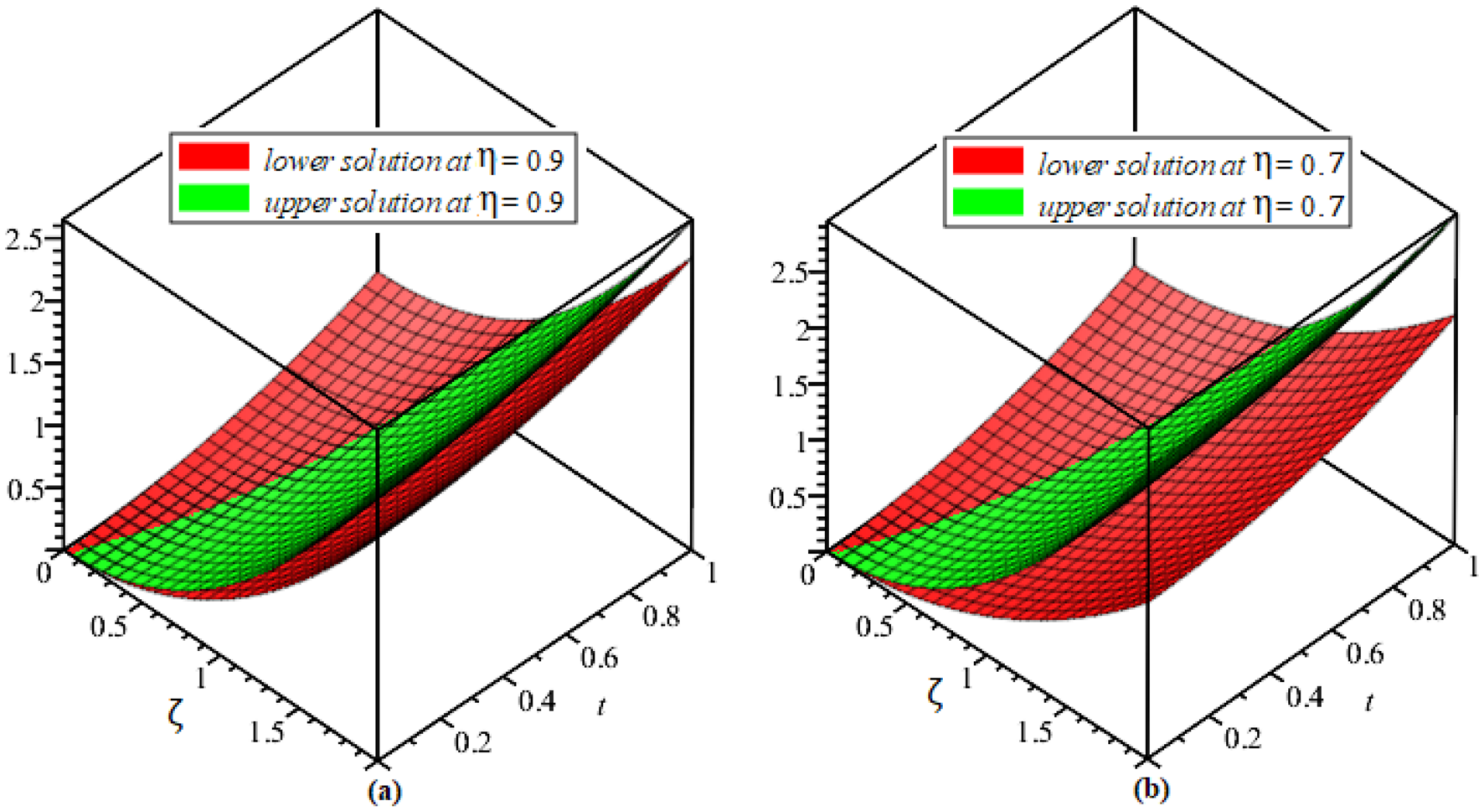
3D plots of the Case 3 lower and upper solutions at (**a**) $\eta$ = 0.7, (**b**) $\eta$ = 0.9 and α = 1.

In this section, the numerical results of the fuzzy FPDEs of Eq. (17) are discussed. In Table 1, the lower and upper solutions are compared for case 1 of problem 4.1 at ζ = 1 and t = 0.1 and various fractional order and also compared with the solution of fuzzy Sawi decomposition method^20^ and the results are found to be identical with fuzzy sawi decomposition method with less computational works. Similarly, In Table 2, the lower and upper solutions are compared for case 3. The 2D and 3D plots are presented to highlight the LRPSM results at different values of parameters. In Fig. 1, the 2D-plots of the Case 1 lower solutions at (a) η = 0.7, t = 0.7, (b) η = 0.4, t = 0.1 and various values of α are presented and, In Fig. 2, the 2D-plots of the Case 1 upper solutions at (a) η = 0.7, t = 0.7, (b) η = 0.4, t = 0.1 and various values of α are presented. Similarly, In Fig. 3 2D-Plots of lower and upper solutions at η, t = 0.7 and α = 0.7. In Fig. 4, 3D-plots of upper and lower solutions are presented. For Case 2, the solution is the same as Case 1. For Case 3, the 2D-plots of lower and upper solutions are presented in Figs. 5 and 6 respectively. 2D-plots of the lower and upper solutions at α = 0.7 are shown in Fig. 7. Similarly, In Fig. 8, 3D-plots for case 3 are presented at α = 1. With the help of FC, we can study and analyze the physical behavior of non-linear problem by simulating and displaying its physical properties. The suggested technique is more suitable and efficient in analyzing complex coupled fractional-order problems. All the numerical calculations are done by Maple 2020.

**Table 1:** Lower and upper solutions comparison of Case 1 of problem 4.1 at ζ = 1 and t = 0.1 and various fractional order.

$\eta$	$\underline{v}{\left(\zeta ,t\right)}_{\alpha =0.8}$	$\overline{v}{\left(\zeta ,t\right)}_{\alpha =0.8}$	$\underline{v}{\left(\zeta ,t\right)}_{\alpha =1}$	$\overline{v}{\left(\zeta ,t\right)}_{\alpha =1}$	$\underline{v}{\left(\zeta ,t\right)}_{\alpha =1} \left[21\right]$	$\overline{v}{\left(\zeta ,t\right)}_{\alpha =1}[21]$
0.1	0.05175702524	0.9833834795	0.050500000	0.95950000	0.050500000	0.95950000
0.2	0.1035140505	0.9316264543	0.101000000	0.90900000	0.101000000	0.90900000
0.3	0.1552710757	0.8798694290	0.151500000	0.85850000	0.151500000	0.85850000
0.4	0.2070281010	0.8281124038	0.202000000	0.80800000	0.202000000	0.80800000
0.5	0.2587851262	0.7763553786	0.252500000	0.75750000	0.252500000	0.75750000
0.6	0.3105421514	0.7245983533	0.303000000	0.70700000	0.303000000	0.70700000
0.7	0.3622991767	0.6728413281	0.353500000	0.65650000	0.353500000	0.65650000
0.8	0.4140562019	0.6210843029	0.404000000	0.60600000	0.404000000	0.60600000
0.9	0.4658132272	0.5693272776	0.454500000	0.55550000	0.454500000	0.55550000
1	0.5175702524	0.5175702524	0.505000000	0.05050000	0.505000000	0.05050000

**Table 2:** Lower and upper solutions comparison of Case 3 of problem 4.1 at ζ = 1 and t = 0.1 and various fractional order.

$\eta$	$\underline{v}{\left(\zeta ,t\right)}_{\alpha =0.8}$	$\overline{v}{\left(\zeta ,t\right)}_{\alpha =0.8}$	$\underline{v}{\left(\zeta ,t\right)}_{\alpha =1}$	$\overline{v}{\left(\zeta ,t\right)}_{\alpha =1}$	$\underline{v}{\left(\zeta ,t\right)}_{\alpha =1}$	$\overline{v}{\left(\zeta ,t\right)}_{\alpha =1}$
0.1	0.08338347954	0.9517570252	0.059500000	0.95050000	0.059500000	0.95050000
0.2	0.1316264543	0.9035140505	0.109000000	0.90100000	0.109000000	0.90100000
0.3	0.1798694290	0.8552710757	0.158500000	0.85150000	0.158500000	0.85150000
0.4	0.2281124038	0.8070281010	0.208000000	0.80200000	0.208000000	0.80200000
0.5	0.2763553786	0.7587851262	0.257500000	0.75250000	0.257500000	0.75250000
0.6	0.3245983533	0.7105421514	0.307000000	0.70300000	0.307000000	0.70300000
0.7	0.3728413281	0.6622991767	0.356500000	0.65400000	0.356500000	0.65400000
0.8	0.4210843029	0.6140562019	0.406000000	0.60600000	0.406000000	0.60600000
0.9	0.4693272776	0.5658132272	0.455500000	0.55450000	0.455500000	0.55450000
1	0.5175702524	0.5175702524	0.505000000	0.50500000	0.505000000	0.50500000

## Conclusion

It is not always easy to find the solutions of FPDEs while using the uncertain experimental data. For this purpose, the solutions of Fuzzy FPDEs are presented to overcome this difficulty. The LRPSM is used to investigate the solutions of some Fuzzy FPDEs. The upper and lower solutions of the targeted problems achieved in a very simple and effective manner. The lower and upper bound solutions are calculated which confirmed the closed contact between them. The accuracy of the suggested method is impressive. The fractional solutions are of higher interest and provide the useful dynamics of the targeted problems. The obtained solutions of the suggested problems are compared Elzaki transform method and Fuzzy Sawi decomposition methods. A very strong agreement of the obtained solutions is confirmed with the solutions of other existing method. The non-linearity is handled in a sophisticated manner as compare to other techniques such as LADM, q-HAM and RPSM. These existing techniques required more calculations to calculate the nonlinearity in each problem by using various polynomials such as Adomian and He’s polynomial. The present method has some limitations depending on the problem’s nature and complexity. For this one reason is the behavior of Laplace transformation over the suggested problems. The suitability of LRPSM with non-singular kernel operator is also a point of investigation. However, the mathematicians are doing their best to control all these issues. Because of utilizing the higher accurate solutions of the fuzzy and fractional concepts and well suitability with Caputo operator, the technique can be modified to solve other higher dimensional and nonlinear problems.

## Data Availability

The datasets used and/or analysed during the current study available from the corresponding author on reasonable request.
